# Chlamydial Infection-Dependent Synthesis of Sphingomyelin as a Novel Anti-Chlamydial Target of Ceramide Mimetic Compounds

**DOI:** 10.3390/ijms232314697

**Published:** 2022-11-24

**Authors:** Keigo Kumagai, Shota Sakai, Masaharu Ueno, Michiyo Kataoka, Shu Kobayashi, Kentaro Hanada

**Affiliations:** 1 Department of Biochemistry and Cell Biology, National Institute of Infectious Diseases, 1-23-1 Toyama, Shinjuku-ku, Tokyo 162-8640, Japan; 2 Department of Natural Science, Graduate School of Advanced Technology and Science, Tokushima University, 2-1 Minami-jousanjima, Tokushima 770-8506, Japan; 3 Department of Pathology, National Institute of Infectious Diseases, 1-23-1 Toyama, Shinjuku-ku, Tokyo 162-8640, Japan; 4 Department of Chemistry, School of Science, University of Tokyo, 7-3-1 Hongo, Bunkyo-ku, Tokyo 110-0033, Japan

**Keywords:** infectious disease, *Chlamydia trachomatis*, bacterial metabolism, antibiotics, ceramide, sphingolipid, inhibitor, stereoselectivity, CERT, sphingomyelin synthase

## Abstract

The obligate intracellular bacterium *Chlamydia trachomatis* is the major causative agent of bacterial sexually transmitted diseases worldwide. In infected cells, the ceramide transport protein (CERT) is recruited to inclusions, where *C. trachomatis* replicates using host-synthesized ceramide. The ceramide is converted to sphingomyelin (SM) by a chlamydial infection-dependent SM synthesis (cidSM-synthesis) pathway, which occurs even in the absence of the SM synthases (SMS)-1 and -2 of host cells. The ceramide mimetic compound (1*R*,3*S*)-HPA-12 and the nonmimetic compound E16A, both of which are potent inhibitors of CERT, repressed the proliferation of *C. trachomatis* in HeLa cells. Unexpectedly, (1*R*,3*R*)-HPA-12, a ceramide mimetic compound that lacks CERT inhibitory activity, also exhibited potent anti-chlamydial activity. Using endogenous SMS-knockout mutant HeLa cells, we revealed that (1*R*,3*R*)-HPA-12 mildly inhibited cidSM-synthesis. In addition, LC-MS analysis revealed that (1*R*,3*R*)-HPA-12 is converted to a phosphocholine-conjugated metabolite in an infection-dependent manner. Imaging analysis with a fluorescent analog of ceramide suggested that cidSM-synthesis occurs in the bacterial bodies and/or inclusions. Collectively, these results suggested that (1*R*,3*R*)-HPA-12 exerts its anti-chlamydia activity not only as an inhibitor of cidSM-synthesis, but also via putative toxic effects of its phosphocholine adduct, which is most likely produced by the cidSM-synthesis route.

## 1. Introduction

*C. trachomatis* is an obligate intracellular bacterium that is the leading cause of both bacterial sexually transmitted diseases worldwide and non-congenital blindness in regions with insufficient sanitation and hygiene [1]. *Chlamydia* spp. have a dimorphic developmental cycle, alternating between a spore-like non-replicative infectious elementary body (EB) and a replicative non-infectious reticulate body (RB). Following cell binding and entry, the EB is sequestered within a specific membranous compartment, termed an inclusion, in which the EB differentiates into the RB by binary fission [2,3]. After multiple divisions, the RB asynchronously begins to differentiate back into the EB; in order to initiate new rounds of infection, the EB is then released from the inclusion to the extracellular space by cell lysis or extrusion [2,3,4].

Most vertebrate organisms produce SM, a phosphocholine-containing sphingolipid. In contrast, no plants, single-cell eukaryotes, or bacteria appear to produce sphingomyelin (SM) [5]. Nevertheless, isolated *C. trachomatis* EBs have been reported to contain SM [6,7] and it has been suggested that this SM is acquired from the host during *C. trachomatis* proliferation. In mammalian cells, the ceramide transport protein (CERT) delivers de novo synthesized ceramide from the endoplasmic reticulum (ER) to the Golgi apparatus. Once in the Golgi apparatus, the ceramide is converted to SM by the following two SM synthases (SMSes): SMS-1 is predominantly localized to the Golgi apparatus, while SMS-2 is mainly localized to the plasma membrane and minorly localized to the Golgi apparatus [8]. CERT is a host-derived factor that is essential for the growth of various *Chlamydia* species, including *C. trachomatis* [9,10], *C. muridarum* [11], and *C. psittaci* [12]. In chlamydia-infected cells, CERT is recruited to the chlamydial inclusions, thereby re-directing newly synthesized ceramide from the ER to the inclusions [9,11]. It has been suggested that the transported ceramide accumulates on the RB membrane [13,14] and is completely converted to SM when *C. trachomatis* differentiates back to the EB [15]. Although host-derived SMS-2 was shown to be recruited to the chlamydial inclusions [11], it was recently reported that a double knock-out of *SGMS1* and *SGMS2* (which encode SMS-1 and SMS-2, respectively) in human cervical carcinoma-derived HeLa cells (hereafter referred to as HeLa∆SMS-1/2 cells) did not affect chlamydial infection or proliferation [10]. Instead, the ceramide to SM converting activity only appeared in *C. trachomatis*-infected HeLa∆SMS-1/2 cells [10], but we could not find any clear orthologs of mammalian SMSes in the *C. trachomatis* genome. In addition, although chemical inhibitors of a biological process are often used to investigate the physiological functions of the biological process, no cidSM-synthesis inhibitors have been reported.

In this study, we found that the (1*R*,3*R*) stereoisomer of HPA-12, a ceramide mimetic compound, exhibited potent anti-chlamydia activity. (1*R*,3*R*)-HPA-12 was found to act as a mild inhibitor of cidSM-synthesis despite its lack of inhibitory activities against the host factors CERT, SMS-1, and SMS-2. Furthermore, we revealed that (1*R*,3*R*)-HPA-12 is efficiently converted to its phosphocholine-conjugated metabolite probably via the cidSM-synthesis route. Our studies with the novel inhibitor (1*R*,3*R*)-HPA-12 provide the first evidence of the physiological function of cidSM-synthesis.

## 2. Results

### 2.1. Repression of C. trachomatis Proliferation in HeLa Cells by CERT Inhibitors

**Figure 1:**
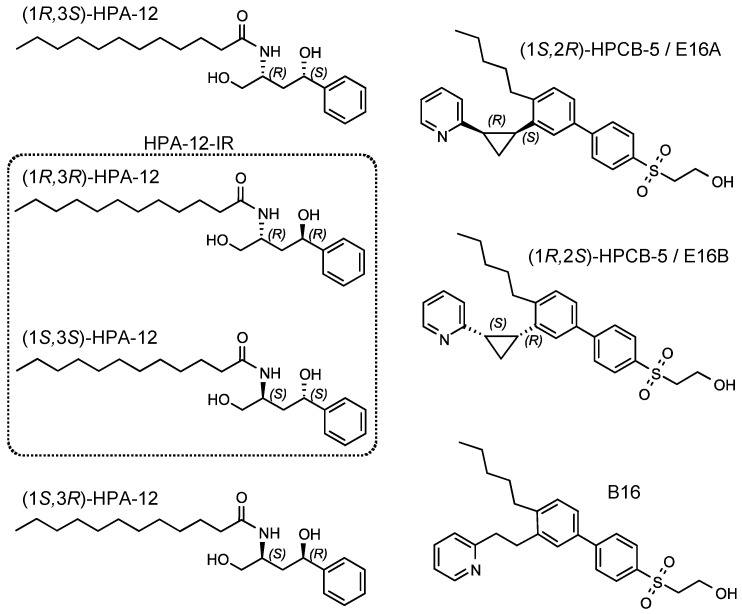
Structures of ceramide mimetic and nonmimetic CERT inhibitors and their relatives. The chemical structure and stereo-configuration of the ceramide mimetic and nonmimetic CERT inhibitors and their relatives used in this study are shown. The rounded rectangle represents the racemic mixture of HPA-12-IR.

**Figure 2:**
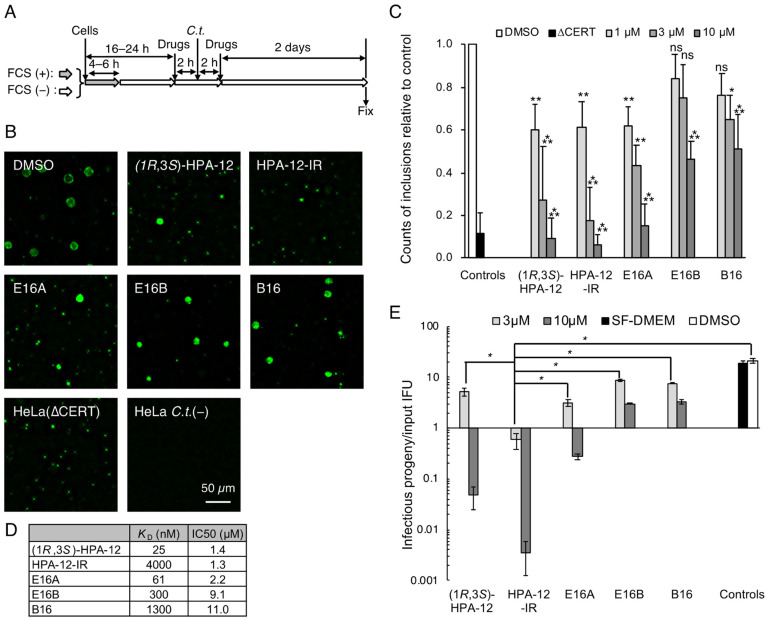
Repression of *C. trachomatis* proliferation by isomers of HPA-12 that possess CERT inhibitory activity and those that lack CERT inhibitory activity. (**A**) Schematic diagram of the experiment designed to measure the IFUs. The cells were maintained in SF-DMEM, pretreated with 10 μM of the indicated compound for 2 h, infected with *C. trachomatis* for 2 h at an MOI of 0.5, and cultured in SF-DMEM containing 10 μM of the compound for 2 days. Next, the cells were fixed and stained with FITC-conjugated anti-chlamydial LPS antibody. (**B**) Immunostaining pattern of chlamydia-infected HeLa cells treated with the compound as described above. The representative fluorescence micrographs of three independent experiments are shown. Scale bar, 50 μm. (**C**) Concentration-dependent decrease in IFUs mediated by the compounds. The cells were treated as described above, except for the use of various concentrations of each compound. In order to calculate the IFUs, FITC-positive spots with an area >50 μm^2^ were counted using ImageJ software. The data represent the mean ± SD of triplicate experiments. Statistically significant values were compared to the DMSO control by Dunnett’s test (* *p* < 0.05; ** *p* < 0.01; *** *p* < 0.001; ns, not significant). (**D**) Summary of the inhibitory effects of the compounds. The *K*_D_ values of the compounds for CERT were reported previously [18]. The IC_50_ values for chlamydial primary IFUs were calculated by logistic regression with ImageJ software using the data shown in panel C. (**E**) Progeny formation assay of chlamydia-infected cells treated with each compound. HeLa cells were infected with *C. trachomatis* and treated with each compound, as described above. At 2 days post-infection, the cells were lysed with sterilized water and fresh confluent HeLa cell monolayers were infected with the lysates. The number of total infectious progeny was divided by the input IFU to generate the reproduction rate. The experiments were performed in triplicate and the data (mean ± SD) are shown with a logarithmic display in this panel. A two-tailed paired Student’s *t*-test with a Bonferroni correction between the groups of cells treated with DMSO and 3 μM of compounds was performed (* *p* < 0.01).

We previously developed two types of potent CERT inhibitors: (1*R*,3*S*)-HPA-12, a ceramide mimetic compound [16,17], and E16A (also known as (1*S*,2*R*)-HPCB-5), a ceramide nonmimetic compound (Figure 1) [18]. HPA-12 has two chiral carbons comprising four stereoisomers (Figure 1); only the (1*R*,3*S*)-configuration exhibits potent CERT inhibition activity [16,19,20,21]. Notably, the initial assigned configuration of active HPA-12 [16,22] was later revised to (1*R*,3*S*) [19,20,21]. A racemic mixture of (1*R*,3*R*)/(1*S*,3*S*)-HPA-12 (hereafter abbreviated as HPA-12-IR) is a control that lacks CERT inhibitory activity (Figure 1 and Figure 2D). E16B (the enantiomer of E16A) and B16 (another ceramide nonmimetic compound) also act as CERT inhibitors but show less inhibitory activity than E16A (Figure 1 and Figure 2D) [18]. In order to test the anti-chlamydial activity of these CERT inhibitors, *C. trachomatis*-infected HeLa cells were exposed to a relatively high concentration (10 μM) of each compound in serum-free Dulbecco’s modified Eagle’s medium (SF-DMEM) and immunostained with FITC-conjugated anti-chlamydial lipopolysaccharide (LPS) antibody (Figure 2A). It is of note that serum-derived lipids might complement the specific lipid requirements of *C. trachomatis* proliferation [23] even when lipid synthesis is blocked. In addition, serum proteins often adsorb various amphipathic compounds and reduce their biological efficacy. A previous study showed that *C. trachomatis* can proliferate in HeLa cells cultured in SF-DMEM [10]. Thus, in order to detect the anti-chlamydial activity of the above compounds with high sensitivity, we used SF-DMEM in this study unless otherwise noted. It was observed that (1*R*,3*S*)-HPA-12 and E16A clearly repressed the inclusion formation to an extent comparable to that observed with genetic disruption of CERT in HeLa cells (hereafter referred to as HeLa∆CERT) (Figure 2B). In contrast, E16B and B16 were less effective at inhibiting inclusion formation than E16A (Figure 2B). In order to compare the effects of the compounds on primary inclusion forming units (IFU) in detail, the cells were treated with various concentrations of each compound and the number of inclusions with an area >50 μm^2^ were counted. The primary IFU in the compound-treated cells was decreased in a dose-dependent manner (Figure 2C). The order of the calculated half-maximal inhibitory concentration (IC_50_) values was consistent with the affinity of these ceramide nonmimetic compounds for CERT (Figure 2D) [18].

### 2.2. Repression of C. trachomatis Proliferation by HPA-12 Isomers Which Lack CERT Inhibitory Activity

Unexpectedly, HPA-12-IR was a potent repressor of inclusion formation, even though we employed it as a negative control (Figure 2B–D). The IC_50_ value showed that the anti-chlamydial activity of HPA-12-IR was comparable to that of (1*R*,3*S*)-HPA-12 and E16A (Figure 2D). By metabolic labeling of HeLa cells with isotope-labeled serine, we confirmed that the HPA-12-IR used in this study had no CERT inhibitory activity. In addition, we found that de novo SM synthesis was not affected by HPA-12-IR, while it was significantly repressed by the CERT inhibitors used in this study (Supplementary Figure S1), being consistent with the findings of previous studies [18].

In the inclusion formation assay, we counted the number of the inclusions with an area >50 μm^2^ as “normally grown inclusions”. However, the size of an inclusion is not necessarily proportional to chlamydial replication and progeny reproduction [24], thus we conducted an infectious progeny formation assay. Lysates prepared from chlamydia-infected HeLa cells in the presence or absence of each compound were added to fresh HeLa cell monolayers, and the numbers of infectious progeny in the lysates were determined. When infected cells were cultured with 3 μM of HPA-12-IR, the values of infectious progeny/input IFU were <1.0 (Figure 2E). HPA-12-IR was the most potent repressor of progeny formation among the five compounds examined. These results indicated that HPA-12-IR inhibits chlamydial propagation by a mechanism distinct from CERT inhibition.

### 2.3. (1R,3R)-HPA-12 Inhibited the Redistribution of a Fluorescence-Labeled Ceramide in the Inclusions

**Figure 3:**
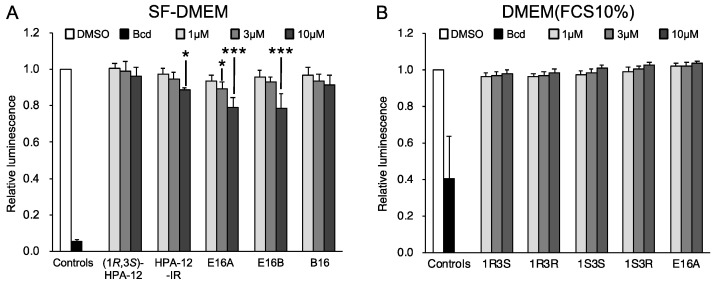
The level of ATP in HeLa cells is not affected by treatment with 3 μM of HPA-12-IR. (**A**) HeLa cells were maintained in SF-DMEM with the indicated compound concentration for 2 days. (**B**) HeLa cells were maintained in DMEM/10% FCS with the indicated compound concentration for 30 h. The levels of ATP in the cells, assayed using the CellTiter-Glo v2 kit, are shown as the relative values compared to the vehicle DMSO-treated cells. The mean values ± SD from three experiments are shown. Dunnett’s test was performed to assess significant differences between compound treatments and the DMSO control. Statistically significant values (* *p* < 0.05; *** *p* < 0.001) are indicated. Bcd: 10 μg/mL of blasticidin.

**Figure 4:**
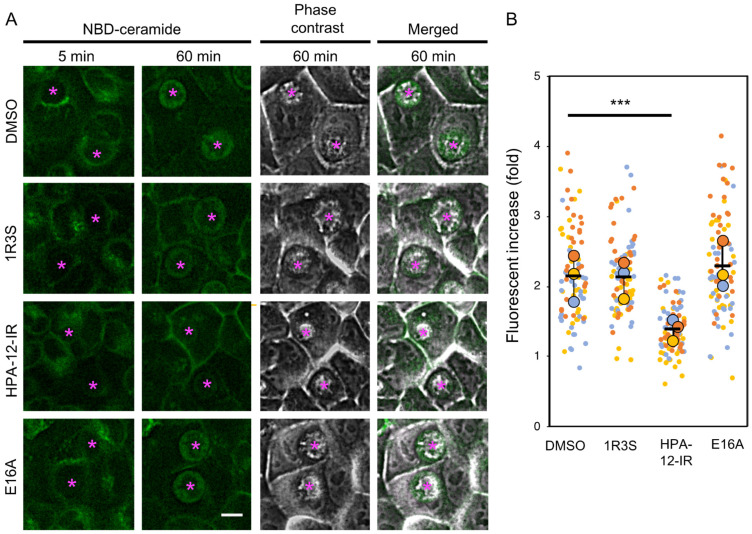
HPA-12-IR inhibited the redistribution of C_6_-NBD-ceramide in the inclusions. (**A**) Chlamydia-infected wild-type HeLa cells were maintained in DMEM/10% FCS for 24 h, then incubated at 4 °C for 30 min in the dark with 5 μM of C_6_-NBD-ceramide in the presence or absence of 10 μM of the indicated compound in serum-free FluoroBrite medium. The temperature was shifted to 37 °C and images of cells in pre-determined fields were obtained at 5 and 60 min by fluorescence and phase-contrast microscopy. The representative fluorescence micrographs of three independent experiments are shown. Asterisks indicate the inclusions. 1R3S: (1*R*,3*S*)-HPA-12. Scale bar, 10 μm. (**B**) The integrated fluorescence intensity of each inclusion at 60 min after the temperature shift to 37 °C was quantified and is shown as the relative values compared to the intensity at 5 min after the temperature shift. The values of each inclusion from three independent experiments are shown in the bee-swarm plot (SuperPlots) [29]. The small symbols indicate the values of each inclusion, and the large symbols indicate the means of the values in each experiment. The results of the three experiments are shown in yellow, blue, and orange, respectively. Statistically significant values obtained by Steel’s multiple comparison test (*** *p* < 0.001) are indicated.

The level of ATP in HeLa cells was not discernibly affected by treatment with 3 μM of HPA-12-IR, although this treatment did inhibit chlamydial progeny formation (Figure 2E and Figure 3A). Thus, the possibility was ruled out that the anti-chlamydial effects of HPA-12-IR were merely due to host cell toxicity. Next, we hypothesized that in chlamydia-infected cells, the ceramide mimetic compound HPA-12-IR might perturb the sphingolipid distribution that is associated with chlamydial proliferation. In order to test this hypothesis, chlamydia-infected wild-type HeLa cells were cultured in DMEM/10% FCS in the presence of each compound followed by labeling with a short-chain ceramide conjugated with the fluorescent dye 4-nitrobenzo-2-oxa-1,3-diazole (NBD) (hereafter referred to as C_6_-NBD-ceramide) for 30 min at 4 °C. At 5 and 60 min after, the temperature of the cultured cells was shifted to 37 °C (the temperature at which C_6_-NBD-ceramide is capable of rapidly and spontaneously transferring between membranes [25]), the cells were observed by fluorescence microscopy. NBD-fluorescence accumulated in the inclusions from mock-treated cells (Figure 4A), as previously reported [15]. Of note, the fluorescence was heterogeneously distributed in the inclusions from the chlamydia-infected wild-type HeLa cells. We found that the fluorescence was high in the peripheral region and low in the central region, which suggested that the fluorescence mainly distributed in the RB that is often abundant near the inclusion membrane and poor in the central region at this life stage [26]. HPA-12-IR significantly reduced the redistribution of the NBD-fluorescence signal into the chlamydial inclusions, while (1*R*,3*S*)-HPA-12 and E16A did not exhibit these effects (Figure 4A,B). C_6_-NBD-ceramide, but not C_6_-NBD-SM, can easily traverse phospholipid membranes [25]. Thus, the accumulation of NBD-fluorescence in a specific organelle is often caused by the conversion of C_6_-NBD-ceramide to its non-translocatable polar metabolite(s) at the luminal side of the organelle [27]. Consistently, 1-*O*-methyl-NBD-ceramide-C_16_, an NBD-ceramide derivative that is incapable of being converted to SM, was recently shown to be minimally accumulated inside chlamydial inclusions [28]. In addition, cidSM-synthesis was recently detected in chlamydia-infected HeLa∆SMS-1/2 cells (10). Thus, the results from our current study together with previous studies led us to the hypothesis that HPA-12-IR inhibits cidSM-synthesis activity, which is presumably capable of converting C_6_-NBD-ceramide to C_6_-NBD-SM in the RBs and/or inclusions.

### 2.4. (1R,3R)-HPA-12 Is an Inhibitor of cidSM-Synthesis

**Figure 5:**
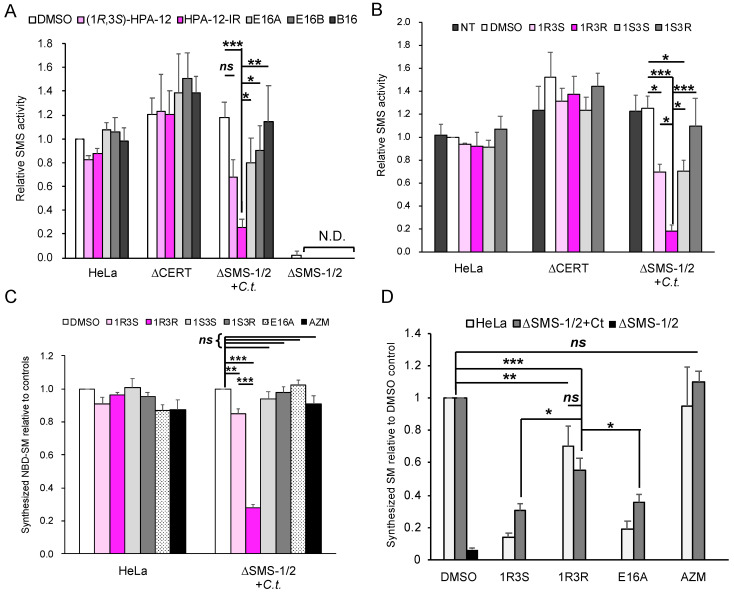
(1*R*,3*R*)-HPA-12 is an inhibitor of cidSM-synthesis. (**A**) The effects of various compounds on the SMS activity in cell lysates. The SMS activity was determined and is shown relative to that from the DMSO-treated lysate fraction from HeLa cells. The mean values ± SD from three experiments are shown. The statistical significance between drug-treated groups in the HeLa∆SMS-1/2+*C.t.* cells was assessed by Tukey’s test and statistically significant values are indicated (* *p* < 0.05; ** *p* < 0.01; *** *p* < 0.001). *C.t*.: *C. trachomatis* serovar L2-infected; N.D.: not determined. (**B**) The effects of various HPA-12 stereoisomers on SMS activity in cell lysates. The SMS activity was determined in the presence or absence of 10 μM of each HPA-12 stereoisomer and is shown as relative to that of the DMSO-treated lysate fraction from HeLa cells. The mean values ± SD from three experiments are shown. Statistical significance between drug-treated groups in the HeLa∆SMS-1/2+*C.t.* cells were assessed by Tukey’s test and statistically significant values are indicated (* *p* < 0.05; *** *p* < 0.001). 1R3S: (1*R*,3*S*)-HPA-12; 1R3R: (1*R*,3*R*)-HPA-12; 1S3S: (1*S*,3*S*)-HPA-12; 1S3R: (1*S*,3*R*)-HPA-12. (**C**) The effects of each compound on the metabolism of C_6_-NBD-ceramide in living cells. HeLa cells or *C. trachomatis*-infected HeLa∆SMS-1/2 cells were incubated with 1 μM of C_6_-NBD-ceramide in the presence or absence of the indicated compound for 45 min at 37 °C. Next, the lipids were extracted and separated by TLC. The levels of C_6_-NBD-SM are shown relative to the level in the DMSO-treated control. Statistical significance between drug-treated groups in the HeLa∆SMS-1/2+*C.t.* cells were assessed by Tukey’s test and statistically significant values are indicated (** *p* < 0.01; *** *p* < 0.001; ns, not significant). AZM: azithromycin. (**D**) The effects of each compound on SM synthesis in living cells. The cells were labeled with [^14^C]serine for 2 h at 37 °C in the presence or absence of 10 μM of the indicated compound. The lipids were extracted and separated by TLC. The relative radioactivity of the synthesized SM in the compound-treated cells normalized to the DMSO control cells are shown, except that the DMSO-treated HeLa∆SMS-1/2 cells was normalized to the DMSO-treated HeLa cells. Statistical significance between the selected groups (HeLa cells treated with DMSO or (1*R*,3*R*)-HPA-12, and chlamydia-infected HeLa∆SMS-1/2 cells treated with (1*R*,3*S*)-HPA-12, (1*R*,3*R*)-HPA-12, E16A, and azithromycin) was assessed by Tukey’s test and statistically significant values are indicated (* *p* < 0.05; ** *p* < 0.01; *** *p* < 0.001; ns, not significant).

In order to test the hypothesis that HPA-12-IR acts as an inhibitor of cidSM-synthesis, cell lysates were prepared from *C. trachomatis*-infected and uninfected HeLa∆SMS-1/2 cells and the effects of each compound on the C_6_-NBD-ceramide to C_6_-NBD-SM conversion activity was examined. The lysates from chlamydia-infected HeLa∆SMS-1/2 cells showed SMS activity at a level comparable with that observed in uninfected wild-type HeLa cells. In contrast, uninfected HeLa∆SMS-1/2 cells showed no discernible SMS activity (Figure 5A). The cidSM-synthesis activity in the HeLa∆SMS-1/2 cell lysate was strongly inhibited by HPA-12-IR, but not by (1*R*,3*S*)-HPA-12, E16A, E16B, or B16 (Figure 5A). HPA-12-IR did not inhibit SMS activity in the uninfected wild-type HeLa cell lysate (Figure 5A). Notably, the level of SMS activity in the HeLa∆CERT cell lysate was comparable with the level observed in the wild-type HeLa cell control lysate (Figure 5A), which indicated that the SMS assay using C_6_-NBD-ceramide as the enzyme substrate is independent of CERT. These results indicated that HPA-12-IR acts as an inhibitor of cidSM-synthesis.

In order to identify the configuration of the HPA-12 stereoisomer that was the most potent inhibitor of cidSM-synthesis, we obtained four newly synthesized HPA-12 stereoisomers (for the quality-check details of these compounds, see Supplementary Figure S2) and analyzed their effects on cidSM-synthesis in vitro. We identified (1*R*,3*R*)-HPA-12 as the most potent inhibitor of cidSM-synthesis (Figure 5B). (1*R*,3*S*)-HPA-12 and (1*S*,3*S*)-HPA-12 showed moderate inhibitory activity against cidSM-synthesis, while (1*S*,3*R*)-HPA-12 lacked cidSM-synthesis inhibitory activity (Figure 5B). None of the HPA-12 stereoisomers inhibited human SMS activity in wild-type HeLa or HeLa∆CERT cells (Figure 5B). In order to test whether (1*R*,3*R*)-HPA-12 can inhibit cidSM-synthesis in living HeLa cells, wild-type HeLa cells and chlamydia-infected HeLa∆SMS-1/2 cells were co-cultured with each HPA-12 stereoisomer and labeled with C_6_-NBD-ceramide for 45 min. The conversion of C_6_-NBD-ceramide into its SM metabolite in the infected HeLa∆SMS-1/2 cells was clearly inhibited by 10 μM of (1*R*,3*R*)-HPA-12, whereas this activity in wild-type HeLa cells was not affected (Figure 5C). Of note, the CERT inhibitor E16A did not affect the conversion of C_6_-NBD-ceramide to its SM metabolite, because C_6_-NBD-ceramide can be spontaneously transferred between membranes [30,31,32]. The possibility that the inhibition of bacterial growth by the compounds caused the decrease in C_6_-NBD-SM synthesis was ruled out because the minimum bactericidal concentration of azithromycin [33], a macrolide antibiotic compound approved for treating chlamydia infection, is 0.5 μg/mL and this concentration did not reduce C_6_-NBD-SM synthesis under our short labeling conditions (Figure 5C).

C_6_-NBD-ceramide is not a natural substrate of SMSes. In order to elucidate whether (1*R*,3*R*)-HPA-12 affects de novo synthesis of natural SM via the cidSM-synthesis pathway, we conducted metabolic labeling experiments with radioisotope-labelled serine which functions as a precursor of newly synthesized natural sphingolipids. Uninfected wild-type HeLa cells or chlamydia-infected HeLa∆SMS-1/2 cells at 24 h post-infection were labeled for 2 h with radioactive serine in the presence of 10 μM of each compound. (1*R*,3*S*)-HPA-12 and E16A inhibited SM synthesis in both cell types because these compounds are potent CERT inhibitors (Figure 5D). (1*R*,3*R*)-HPA-12, which lacks CERT inhibitory activity, repressed SM synthesis in both cell types although the efficiency was less than that observed with (1*R*,3*S*)-HPA-12 and E16A (Figure 5D). Collectively, these results indicated that (1*R*,3*R*)-HPA-12 mildly represses the de novo SM synthesis via the cidSM-synthesis pathway.

### 2.5. The Anti-Chlamydial Effect of (1R,3R)-HPA-12 Is Partially Correlated with Inhibition of cidSM-Synthesis

**Figure 6:**
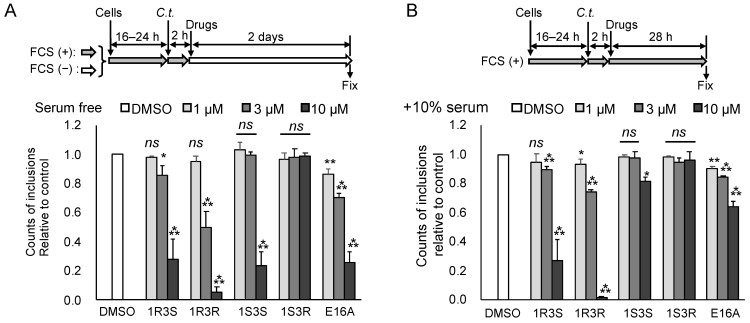
The anti-chlamydial activity of (1*R*,3*R*)-HPA-12 is partially correlated with the inhibition of cidSM-synthesis. (**A**) The anti-chlamydial activity of HPA-12 stereoisomers and E16A under serum-free conditions. HeLa cells were infected with *C. trachomatis* in DMEM/10% FCS for 2 h at an MOI of 0.5 and were maintained in SF-DMEM containing various concentrations of each compound for 2 days, as summarized in the diagram (**upper** panel). After fixing the cells, the inclusions were stained with FITC-conjugated anti-chlamydial LPS antibody, and FITC-positive spots with an area >50 μm^2^ were counted. The number of FITC-positive spots are presented relative to the values counted in the DMSO-treated control cells (**lower** panel). Statistically significant values assessed by Dunnett’s test relative to the DMSO control are indicated (* *p* < 0.05; ** *p* < 0.01; *** *p* < 0.001; ns, not significant). The mean values ± SD from three experiments are shown. (**B**) The anti-chlamydial activity of the compounds under serum-containing conditions. The experimental setup was the same as that for the serum-free conditions, except that the cells were cultured with each compound in DMEM/10% FCS for 30 h post-infection (**upper** panel). Statistically significant values assessed by Dunnett’s test are indicated (* *p* < 0.05; ** *p* < 0.01; *** *p* < 0.001; ns, not significant). The mean values ± SD from three experiments are shown (**lower** panel).

In order to determine whether the inhibition of cidSM-synthesis is related to the anti-chlamydial effect, we estimated the anti-chlamydial activities of the HPA-12 stereoisomers and E16A using a primary inclusion formation assay. Wild-type HeLa cell monolayers were infected with *C. trachomatis* in a normal culture medium (DMEM/10% FCS) for 2 h, and after washing the cell monolayers, the infected HeLa cells were cultured in SF-DMEM for 2 days or in DMEM/10% FCS for 28 h in the presence or absence of each compound (Figure 6A,B). (1*R*,3*S*)-HPA-12, (1*R*,3*R*)-HPA-12, and E16A (10 μM each) inhibited primary inclusion formation in SF-DMEM (Figure 6A) and in DMEM/10% FCS (Figure 6B). (1*S*,3*S*)-HPA-12 also inhibited primary inclusion formation, but only in SF-DMEM (Figure 6A). The anti-chlamydial effects of each compound were reduced in the presence of serum (Figure 6A,B). We also performed an experiment with the chlamydial strain L2/434/Bu using the same method as described for Figure 6B and obtained similar results (Supplementary Figure S3). The fact that these compounds inhibited chlamydial growth after the chlamydial infection indicated that these compounds do not affect *C. trachomatis* cell binding and instead suppress a post-binding step(s). The *C. trachomatis* growth inhibition levels mediated by these compounds were partially correlated with the repression of cidSM-synthesis (Figure 5C and Figure 6A,B). However, it is unlikely that the inhibition of *C. trachomatis* growth is ascribed solely to the repression of cidSM-synthesis because the most potent anti-chlamydial compound (1*R*,3*R*)-HPA-12 was only a moderate inhibitor of cidSM-synthesis (Figure 5D). Based on these results, we decided to examine the possibility that the anti-chlamydial activity of (1*R*,3*R*)-HPA-12 is due to bacterial damage induced by an unknown metabolite(s) derived from (1*R*,3*R*)-HPA-12.

### 2.6. Phospholcholine-Conjugated (1R,3R)-HPA-12, Produced by the cidSM-Synthesis Pathway, Is Related to Anti-Chlamydial Activity

**Figure 7:**
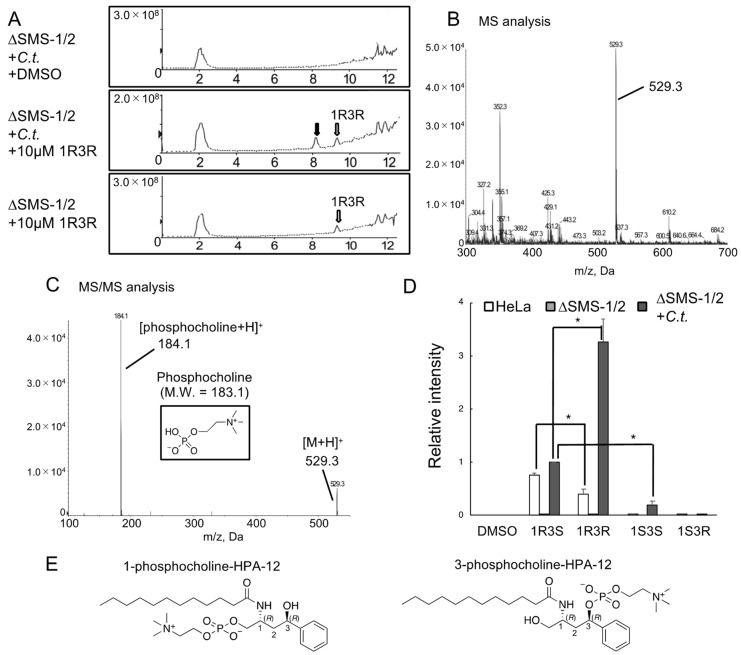
Phosphocholine-conjugated (1*R*,3*R*)-HPA-12 produced by cidSM-synthesis. (**A**) Total ion chromatograms from single mass spectrometry (in the range of *m*/*z* = 300–700) of the methanol extracts from cells treated with 10 μM of each compound. White arrows denote peaks that appeared dependent on the (1*R*,3*R*)-HPA-12 addition; the black arrow indicates a peak that appeared dependent on both chlamydial infection and the (1*R*,3*R*)-HPA-12 addition. (**B**) Mass spectra around the black arrow (retention time of 7–9 min) in Figure 7A of the chlamydia-infected HeLa∆SMS-1/2 cells. A signal close to the mass of phosphocholine-conjugated HPA-12 (PC-HPA-12, 529.3) was observed. (**C**) LC-MS/MS spectra of the precursor ion *m*/*z* 529.3. A signal corresponding to the mass of protonated cholinephosphate was observed. (**D**) The ratios of PC-HPA-12/d7-cholesterol in the indicated cells treated with the stereoisomers of HPA-12. The cells were treated with 3 μM of each compound and the extracted lipids were analyzed by LC-MS/MS in the MRM mode. The data are shown as the mean ± SD from three independent experiments and a two-tailed paired Student’s *t*-test with a Bonferroni correction was performed (* *p* < 0.0167). (**E**) The supposed chemical structures of PC-HPA-12. The systematic chemical name of the compound HPA-12 is *N*-(3-hydroxy-1-hydroxymethyl-3-phenylpropyl)dodecanamide (16). Thus, 1-phosphocholine-HPA-12 means a derivative of HPA-12 with a phosphocholine adduct at the 1-hydroxymethyl group.

In order to test the possibility that the anti-chlamydial activity of (1*R*,3*R*)-HPA-12 is due to an unknown metabolite(s) derived from (1*R*,3*R*)-HPA-12, we treated uninfected or chlamydia-infected HeLa∆SMS-1/2 cells with each compound (including four stereoisomers of HPA-12) and the lipid extracts were analyzed by liquid chromatography-tandem mass spectrometry (LC-MS/MS) analysis. A peak derived from (1*R*,3*R*)-HPA-12 was detected at a retention time of ~9 min (lower panel of Figure 7A). A novel peak appeared at a retention time of ~8 min dependent on chlamydial infection in the cells treated with 10 μM of (1*R*,3*R*)-HPA-12 (middle panel of Figure 7A). The novel peak was mainly composed of a signal that is close to the mass of protonated phosphocholine-conjugated (PC) HPA-12 (PC-HPA-12^+^, *m*/*z* = 529.3) (Figure 7B). MS/MS analysis of the ion of *m*/*z* = 529.3 identified a product ion of *m*/*z* = 184.1, which corresponded to protonated phosphocholine (Figure 7C). Thus, we identified the signal of *m*/*z* = 529.4 as PC-HPA-12, although it was unknown which hydroxy group (1-OH or 3-OH) harbored the conjugated phosphocholine (Figure 7E). We tried to identify phosphocholine-conjugated E16A by the same approach but could not find a clear signal corresponding to it.

Next, we compared the production of PC-HPA-12 derived from four HPA-12 stereoisomers. Uninfected wild-type HeLa or HeLa∆SMS-1/2 cells and chlamydia-infected HeLa∆SMS-1/2 cells were independently treated with 3 μM of each HPA-12 stereoisomer for 4 h and the extracted lipids were analyzed by LC-MS/MS with the multiple reaction monitoring (MRM) mode. Wild-type HeLa and chlamydia-infected HeLa∆SMS-1/2 cells produced PC-HPA-12, while uninfected HeLa∆SMS-1/2 cells did not produce PC-HPA-12 (Figure 7D), indicating that the production of PC-HPA-12 is dependent on human SMSes and chlamydial infection.

Among the four stereoisomers, (1*R*,3*S*)-HPA-12 was most efficiently converted to its phosphocholine adduct in uninfected wild-type HeLa cells. In contrast, (1*R*,3*R*)-HPA-12 was most efficiently converted in chlamydia-infected HeLa∆SMS-1/2 cells. In chlamydia-infected HeLa∆SMS-1/2 cells, the amount of produced PC-HPA-12 was more than 4-fold higher for (1*R*,3*R*)-HPA-12 than for (1*R*,3*S*)-HPA-12 (Figure 7D). During chlamydial infection, the amounts of PC-HPA-12 produced from the four HPA-12 stereoisomers were well correlated with the anti-chlamydial activity (Figure 6 and Figure 7D). These results suggested that the anti-chlamydial effect of the HPA-12 stereoisomers is relevant to CERT inhibition, the production of PC-HPA-12, and the inhibition of cidSM-synthesis.

Notably, among the four HPA-12 stereoisomers, the more efficiently a compound inhibited cidSM-synthesis, the more efficiently it was converted to its phosphocholine adduct in infected HeLa∆SMS-1/2 cells (Figure 6 and Figure 7). These results suggested that (1*R*,3*S*)-HPA-12 and (1*R*,3*R*)-HPA-12 were converted into PC-HPA-12 forms via the cidSM-synthesis route as competitive reactive ligands. This interpretation is consistent with the result that (1*R*,3*S*)-HPA-12, which could be converted to PC-HPA-12 by chlamydial infection, showed a more potent anti-chlamydial activity than E16A, despite their comparable CERT inhibitory activities (Figure 6 and Figure 7D).

### 2.7. Retardation of RB-to-EB Maturation Induced by (1R,3R)-HPA-12 Treatment

**Figure 8:**
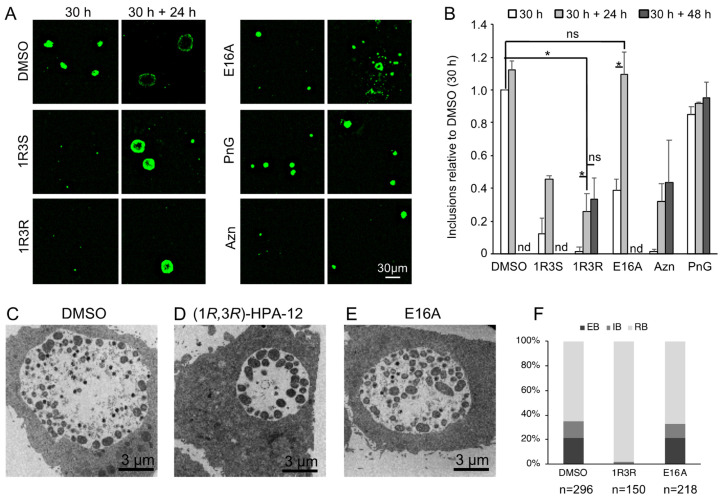
Chlamydial changes induced by treatment with (1*R*,3*R*)-HPA-12. (**A**,**B**) The growth recovery after compound treatment. (**A**) Immunostaining pattern of chlamydia-infected cells. Chlamydia-infected HeLa cells were maintained in DMEM/10% FCS containing 10 μM of (1*R*,3*S*)-HPA-12, 10 μM of (1*R*,3*R*)-HPA-12, 10 μM of E16A, 330 U/mL penicillin G (PnG), or 5 ng/mL azithromycin for 30 h. The medium was then replaced with DMEM/10% FCS and the cells were maintained for an additional 24 or 48 h. Three independent experiments were performed, and representative fluorescence micrographs are shown. Scale bar, 30 μm. (**B**) The number of inclusions from compound-treated cells were normalized to that from DMSO-treated cells at 30 h and the mean values ± SD from three experiments are shown. A Student’s *t*-test with a Bonferroni correction was performed (* *p* < 0.01; ns, not significant; nd, not determined). (**C**–**F**) TEM analysis of chlamydial inclusions. Chlamydia-infected HeLa cells were maintained in DMEM/10% FCS in the presence of (**C**) DMSO, (**D**) 5 μM of (1*R*,3*R*)-HPA-12, or (**E**) 5 μM of E16A for 30 h followed by fixation for TEM analysis. Scale bar, 3 μm. (**F**) Distribution of the developmental phase of *C. trachomatis*. The chlamydial particles classified as an elementary body (EB), a reticulate body (RB), and an intermediate body (IB) were counted and are shown as percentages of the total counts. The data are representative of two independent experiments.

In order to examine whether (1*R*,3*R*)-HPA-12 affects the bacterial proliferation cycle in host cells, we examined chlamydial growth after each compound was removed. Chlamydia-infected cells were divided into three groups and treated with 10 μM of each compound for 30 h. Subsequently, one group was fixed immediately, and the other groups were fixed after drug-free incubation for 24 h or 48 h in DMEM/10% FCS. The fixed cells were immunostained with a fluorescence-labeled anti-chlamydial LPS antibody and observed by fluorescence microscopy (Figure 8A). The number of inclusions decreased upon treatment with 10 μM of (1*R*,3*R*)-HPA-12 for 30 h and recovered to ~30% of the loading dose (mock-treated chlamydia-infected cells at 30 h) after the drug-free incubation for 24 h. These results indicated that a minor population of *C. trachomatis* survived in small inclusions (<50 μm^2^), but most of the population died after drug treatment (Figure 8B). Drug-free incubation for 24 h was sufficient for the recovery of inclusion formation because extended drug-free incubation up to 48 h did not further increase the number of inclusions (Figure 8B). The decreased number of inclusions in cells treated with 10 μM of E16A was completely recovered to the loading dose level by a drug-free incubation of 24 h (Figure 8B). This result suggested that 10 μM of E16A was not directly cytotoxic against *C. trachomatis*.

Next, we performed transmission electron microscopy (TEM) to analyze the effects of inhibition of both cidSM-synthesis and host CERT activity upon treatment with (1*R*,3*R*)-HPA-12 or E16A. Chlamydia-infected wild-type HeLa cells were maintained for 30 h in DMEM/10% FCS containing 5 μM of each compound (the conditions used did not completely block the proliferation of the bacteria). Morphological analysis revealed that the RB form of *C. trachomatis* started to differentiate back to the EB form when treated with either E16A or the vehicle DMSO control (Figure 8C,E,F). In contrast, when treated with (1*R*,3*R*)-HPA-12, the bacteria residing in the inclusions could not differentiate to the EB form and retained an RB-like morphology, which was slightly larger and darker than the normal RB form detected in the vehicle DMSO control (Figure 8C,D,F). These results suggested that the majority of *C. trachomatis* is eliminated by (1*R*,3*R*)-HPA-12 treatment but a minor population survives in the small inclusions wherein RB-to-EB maturation is delayed or compromised.

## 3. Discussion

In the present study, we verified that both ceramide mimetic and nonmimetic inhibitors of CERT (i.e., (1*R*,3*S*)-HPA-12 and E16A, respectively) exhibited anti-chlamydial activity in cell culture, consistent with previous studies showing that CERT is required for the proliferation of *C. trachomatis* [9,10], *C. psittaci* [12], and *C. muridarum* [11]. In addition, we found that (1*R*,3*R*)-HPA-12, a stereoisomer that lacks CERT inhibitory activity, unexpectedly has potent anti-chlamydial activity. Our analysis suggested that the anti-chlamydia activity is ascribed to at least two mechanisms: (1) (1*R*,3*R*)-HPA-12 inhibits cidSM-synthesis as a competitive substrate of the putative enzyme; and (2) the phosphocholine adduct of (1*R*,3*R*)-HPA-12, which is efficiently produced by the cidSM-synthesis route, possibly interferes with bacterial proliferation in host cells.

Our LC-MS/MS analysis also indicated that (1*R*,3*S*)-HPA-12, which has been widely used as a CERT inhibitor [16,34,35,36], is converted to its phosphocholine-conjugated metabolite presumably via the cidSM-synthesis pathway and also by host SMSes (Figure 7D). Based on these data, we here suggested that (1*R*,3*S*)-HPA-12 exerts its anti-chlamydia activity not only as an inhibitor of CERT or cidSM-synthesis but also via putative toxic effects of its phosphocholine adduct. Of note, we do not deny the possibility that (1*R*,3*S*)-HPA-12 and/or (1*R*,3*R*)-HPA-12 are also converted to other unknown toxic metabolites, for instance, their deacylated forms, at present although their possible metabolites other than the phosphocholine-adducts were not discernibly detected under MS analysis conditions we used. Considering our new findings and insights, we would like to alert fellow researchers that utilize (1*R*,3*S*)-HPA-12 in their studies, that the phosphocholine adduct of (1*R*,3*S*)-HPA-12 or unknown metabolites may exert unwanted side effects independent of CERT inhibition. From this perspective, E16A may be a better CERT inhibitor than (1*R*,3*S*)-HPA-12 although a more efficient method to chemically synthesize E16A will be required for supplying the global demand of this drug. A recent study showed that treatment with the CERT inhibitor HPA-12 produced more pronounced effects on the formation of *C. psittaci* infectious progeny, compared with CERT gene disruption in host cells [12]. This discrepancy may be explained by a metabolite of (1*R*,3*S*)-HPA-12 that has a negative impact(s) on *C. psittaci* proliferation.

Image analysis with C_6_-NBD-ceramide in infected cells suggested that the cidSM-synthesis occurs in the RBs and/or inclusions. These data implied that the enzyme responsible for cidSM-synthesis is encoded by the *C. trachomatis* genome, although the gene encoding the putative chlamydia SMS remains unknown. Despite our extensive searches for chlamydia SMS(es), there is no clear ortholog of mammalian SMS in the genomes from organisms belonging to the *Chlamydia* and *Chlamydophila* genera. The stereochemistry of natural ceramide more closely resembles (1*R*,3*S*)-HPA-12 rather than (1*R*,3*R*)-HPA-12 [21]. Interestingly, the phosphocholine adduct of (1*R*,3*S*)-HPA-12 is produced in uninfected wild-type HeLa cells and infected HeLa∆SMS-1/2 cells to almost the same extent. In contrast, the phosphocholine adduct of (1*R*,3*R*)-HPA-12 is produced in much greater amounts in infected HeLa∆SMS-1/2 cells compared to uninfected wild-type HeLa cells (Figure 7D). These results suggested that there are considerable differences in the substrate selectivity between human SMSes and the putative chlamydia SMS. Thus, we hypothesize that the putative chlamydia SMS is structurally distinct from known SMSes.

Phylogenetic analyses suggest that SM and its close relatives only occur in multicellular animals although plants, unicellular eukaryotes, and some bacteria produce non-SM sphingolipids [5]. However, *C. trachomatis* EBs contain SM, and their lipid composition is quite similar to that of mammalian host cells [37]. This has been explained by the suggestion that *Chlamydia* spp. acquire host-synthesized lipids while proliferating in inclusions. However, our previous and current studies have indicated that *C. trachomatis* exploits host-synthesized ceramide in a CERT-dependent manner and converts ceramide to SM via the cidSM-synthesis pathway. Thus, SM synthesized via this pathway, but not via the host SMS-1 nor SMS-2 routes, may be crucial for chlamydial differentiation in inclusions.

The phosphocholine adducts of the HPA-12 stereoisomers are chemically amphipathic and presumably behave as membrane-damaging detergent-like compounds. Importantly, (1*R*,3*R*)-HPA-12, but not the CERT inhibitor (1*R*,3*S*)-HPA-12, is efficiently converted to its phosphocholine-conjugated metabolite via the cidSM-synthesis route. As discussed above, PC-HPA-12 may be specifically produced in the RBs and/or inclusions. If this is the case, PC-HPA-12 may accumulate in the RBs and/or inclusions at membrane-damaging levels, thereby selectively exhibiting detrimental impacts on the RBs and chlamydia-infected cells even though PC-HPA-12 itself is a non-specific membrane disrupter. Therefore, (1*R*,3*R*)-HPA-12 might serve as an excellent seed compound for new type(s) of anti-chlamydial drugs.

TEM analysis revealed that *C. trachomatis* RBs treated with (1*R*,3*R*)-HPA-12 appeared larger and darker than control DMSO-treated *C. trachomatis* EBs. Abdelrahman et al. reported that BODIPY™-SM (derived from BODIPY™ FL C_5_-ceramide) is enriched at the septum membrane between a mother and daughter cell [13]. Thus, PC-HPA-12 may impair the cell division process of *C. trachomatis*. Inhibition of cell division by PC-HPA-12 may induce swelling of the bacterial body of *C. trachomatis*, which may prolong the duration of RB-to-EB conversion because only RBs below a specific size threshold can convert into EBs [26]. The effects of (1*R*,3*R*)-HPA-12 on the releasing process of *C. trachomatis* was not analyzed because (1*R*,3*R*)-HPA-12 delayed RB-to-EB differentiation, which has a critical impact on the releasing process. The precise mechanism of PC-HPA-12 cytotoxicity against *C. trachomatis* will be clarified in future studies.

## 4. Materials and Methods

### 4.1. Materials

Azithromycin and *N*-[7-(4-nitrobenzo-2-oxa-1,3-diazole)]-6-aminocaproyl-d-*erythro*-sphingosine (C_6_-NBD-ceramide) were purchased from Cayman Chemical (Ann Arbor, MI, USA). In order to generate stock solutions, these compounds were dissolved in DMSO (the ultra-pure grade Hybri-Max™, Sigma-Aldrich, St. Louis, MO, USA) and were stored at −20 °C until use. l-[^14^C(U)]serine was obtained from Moravek (Brea, CA, USA). D7-cholesterol was purchased from Avanti Polar Lipids (Alabaster, AL, USA). FITC-conjugated mouse anti-chlamydial LPS antibody was purchased from Denka Seiken (Tokyo, Japan). DMEM with high glucose and cycloheximide was obtained from FUJIFILM Wako (Osaka, Japan). The TLC plates (Silica gel 60) were purchased from Merck (Darmstadt, Germany).

### 4.2. Cell Culture and Propagation of C. trachomatis

HeLa cells (ATCC^®^ CCL-2, RRID: CVCL_0030) obtained from the American Type Culture Collection (ATCC, Manassas, VA, USA) were used as wild-type HeLa cells in this study. HeLa∆CERT and HeLa∆SMS-1/2 cells were previously established in our laboratory [10,38]. These cells were maintained in DMEM with 10% heat-inactivated fetal calf serum (FCS) at 37 °C in a humidified atmosphere containing 5% CO_2_ and were passaged every three days. When necessary, DMEM containing 0.1% of the Insulin-Transferrin-Sodium Selenite Supplement solution (Roche, Basel, Switzerland) (final concentrations: 5 μg/mL insulin, 5 μg/mL transferrin, and 5 ng/mL sodium selenite) as well as 1% of Lipid Mixture 1 (Sigma-Aldrich) was used as a serum-free culture medium (SF-DMEM). *C. trachomatis* serovar L2 (strain T’ang (TRIC/China/Peking-2/OTf)) (ATCC^®^ VR-577) was purchased from ATCC and L2 (L2/434/Bu) was a kind gift from Dr. Shuji Ando (Department of Virology I, National Institute of Infectious Diseases, Tokyo, Japan) [39]. Both strains were propagated as described previously [10].

### 4.3. Plasmids

The samples of (1*R*,3*S*)-HPA-12, HPA-12-IR, E16A, E16B, and B16 were obtained from laboratory stocks that have been previously chemically synthesized [18,20,22]. Four stereoisomers of HPA-12, synthesized as previously described [19], were a generous gift from Dr. Kouichi Uoto (Daiichi Sankyo Co., Ltd., Tokyo, Japan). There are restrictions on the availability of the HPA-12 stereoisomers, as well as E16A, E16B, and B16 due to the limited amounts of these compounds we have at present.

### 4.4. Primary Inclusion Formation Assay

Wild-type HeLa or HeLa∆CERT cells were seeded in DMEM supplemented with 10% FCS (DMEM/10% FCS) to form confluent cell monolayers in 48-well plates to prepare the cells for chlamydial infection. If necessary, the medium was replaced with SF-DMEM 14 to 18 h prior to infection to minimize carryover of the serum. The cells were pretreated with each compound in SF-DMEM for 2 h prior to infection. The cells were infected with *C. trachomatis* for 2 h at a multiplicity of infection (MOI) of 0.5 for SF-DMEM or an MOI of 0.2 for DMEM/10% FCS. The cells were washed once with culture medium, and the medium was replaced with fresh compound-containing SF-DMEM or DMEM/10% FCS containing the compound. The cells were then incubated for 48 h in SF-DMEM or 30 h in DMEM/10% FCS. When growth recovery from anti-bacterial agents were examined, the compound-treated HeLa cells were washed once with DMEM/10% FCS and maintained in DMEM/10% FCS for 24 h. After removing the medium, the cells were fixed and permeabilized by incubation in prechilled methanol at −20 °C for 10 min. Next, the cells were washed three times with PBS. The fixed and permeabilized cells were stained with FITC-conjugated anti-chlamydial LPS antibody (diluted 100-fold in PBS) at 37 °C for 1 h followed by three washes with degassed PBS. The stained cells were observed under a fluorescence microscope (BZ-X710; Keyence, Osaka, Japan) and the number of inclusions with an area >50 μm^2^ was counted using ImageJ software [40]. When primary inclusion formation was estimated without drug pretreatment, HeLa cells were cultured to form confluent cell monolayers in DMEM/10% FCS and infected with *C. trachomatis* for 2 h. The medium was replaced with the indicated fresh medium containing the compound, and the cells were incubated for 30 h in DMEM/10% FCS or 48 h in SF-DMEM. The cells were fixed and stained as described above.

### 4.5. Progeny Formation Assay

As described in the primary inclusion formation assay section, confluent HeLa cell monolayers grown on 48-well plates were pretreated with each compound in SF-DMEM, infected with *C. trachomatis*, and maintained in the presence of the same compound for 48 h. In order to estimate the primary IFUs, a portion of the chlamydia-infected HeLa cells were fixed by methanol as described above and stored at 4 °C until use. The other HeLa cells were incubated in 200 μL of sterilized water for 5 min at 25 °C and were lysed by pipetting. The lysate (2–50 μL) was used to infect fresh confluent HeLa cell monolayers in DMEM/10% FCS in the presence of 1 μg/mL of cycloheximide. The cells were then fixed and stained as described in the primary inclusion formation assay section.

### 4.6. Cell Growth Assay

HeLa cells were maintained in 100 μL of DMEM/10% FCS in 96-well plates. When SF-DMEM was used, the medium was replaced with SF-DMEM for 14–18 h prior to compound treatment. The confluent HeLa cell monolayers were pretreated with each compound in SF-DMEM or DMEM/10% FCS for 2 h, washed with PBS, and maintained in 100 μL of fresh SF-DMEM containing the compound for 48 h. The cellular ATP levels were quantified using the CellTiter-Glo^®^ 2.0 Assay kit (Promega, Madison, WI, USA), according to the manufacturer’s standard protocol.

### 4.7. SMS Assay

HeLa, HeLa∆CERT, and HeLa∆SMS-1/2 cells were maintained in 100 mm dishes in SF-DMEM for 16 h. The cells were mock-infected or infected with *C. trachomatis* at an MOI of 2.0 for 2 h, and maintained in SF-DMEM for 48 h. The cells were scraped into reaction buffer (20 mM HEPES-KOH (pH 7.0), 15 mM KCl, 5 mM NaCl, 1 mM EDTA, 10 mM MnCl_2_, 300 mM sucrose, and one tablet of the cOmplete EDTA-free Protease Inhibitor Cocktail (Roche)/50 mL of the cell suspension) [41] and were lysed by sonication. The protein concentration of the lysate fractions was determined using the BCA Protein Assay kit (Thermo Fisher, Waltham, MA, USA), and aliquots of the lysates were stored at −80 °C until use. The lysates (10 μg protein/assay) were incubated in 100 μL of the reaction buffer at 37 °C for 15 min in the presence of C_6_-NBD-ceramide (0.5 μM) and either the compound (10 μM) or DMSO (0.5%). The lipids were extracted using the Bligh and Dyer method [42] followed by separation on TLC plates (solvent system: methyl acetate/n-propanol/chloroform/methanol/0.25% KCl = 25/25/25/10/9, *v*/*v*). The separated lipids were analyzed with an image analyzer (Typhoon™ FLA7000, GE Healthcare Life Science, Chicago, IL, USA) and statistical analysis was performed using the EZR software (R-based software for statistical analysis) [43].

### 4.8. Metabolic Labeling and Live-Cell Imaging with C6-NBD-Ceramide

Metabolic labeling was performed as follows: HeLa and HeLa∆SMS-1/2 cells were maintained in 500 μL of DMEM/10% FCS in 24-well plates for 16 to 20 h to form a subconfluent cell monolayer. HeLa∆SMS-1/2 cells were infected with *C. trachomatis* at an MOI of 2.0 for 2 h. For uninfected controls, HeLa cells were mock-infected. The cells were washed once with DMEM/10% FCS and maintained in 500 μL of DMEM/10% FCS for 24 h. The cells were then pretreated with 10 μM of the compound in 250 μL of SF-DMEM for 10 min, followed by labeling for 45 min at 37 °C with 250 μL of SF-DMEM containing 1 μM C_6_-NBD-ceramide and 10 μM of the compound. Next, the cells were lysed with 200 μL of 0.1% SDS to terminate the metabolic labeling. The viscosity of the 0.1% SDS-treated cell lysates was increased due to chromosomal DNA. Thus, the viscosity of the lysates was reduced by sonication in ice water using a bath-type sonicator (Elestein 07-01, Elekon Science Co. Ltd., Chiba, Japan). The lipids from the lysates were extracted and analyzed as described in the SMS assay section. Live-cell imaging with C_6_-NBD-ceramide was performed as follows: Subconfluent HeLa cell monolayers were generated by maintaining the cells in 300 μL of DMEM/10% FCS in an 8-well chamber slide (ibidi GmbH, Martinsried, Germany). The cells were then infected with *C. trachomatis* at an MOI of 2.0 for 2 h, washed once with DMEM/10% FCS, and maintained in 300 μL of DMEM/10% FCS for 24 h. Next, the cells were pretreated for 5 min with 200 μL of SF-DMEM containing 10 μM of the compound. Subsequently, the culture medium was replaced with 200 μL of prechilled SF-DMEM containing 10 μM of the compound and 5 μM of NBD-ceramide. After the cells were incubated on ice for 30 min in the dark, the culture medium was replaced with 200 μL of prewarmed DMEM/10% FCS containing 10 μM of the compound. The cells were incubated at 37 °C in an atmosphere containing 5% CO_2_ in a chamber of the fluorescence microscope BZ-X710 (Keyence), and the cells were observed at 5, 30, 60, and 120 min following the replacement with the prewarmed medium. NBD-derived fluorescence signals were quantified using the ImageJ and EZR software. In addition, the EZR software was used to draw boxplots and perform statistical analyses [43].

### 4.9. Metabolic Labeling with [^14^C]Serine

Cell culture and chlamydial infection were performed as described in the metabolic labeling and live-cell imaging with C_6_-NBD-ceramide section except we grew the cells in 6-well plates with 2 mL of medium. At 24 h post-infection, the cells were pretreated for 30 min with 3 μM of the compound in 1.5 mL of SF-DMEM. The cells were then labeled for 2 h at 37 °C in the presence of 450 nCi of [^14^C]serine in the medium. Next, the cells were lysed with 300 μL of 0.1% SDS. The lipids in the lysates were extracted and analyzed as described in the SMS assay section.

### 4.10. LC-MS Analysis

Cell culture and chlamydial infection were performed as described in the metabolic labeling and live-cell imaging with C_6_-NBD-ceramide section except we grew the cells in 1 mL of DMEM/10% FCS in 12-well plates. At 24 h post-infection, the cells were pretreated for 4 h with 3 μM or 10 μM of the compound in 500 μL of DMEM/10% FCS. The cells were washed once with 500 μL of PBS and harvested in 100 μL of PBS by scraping. The cell suspension was mixed with 400 μL of methanol containing 500 pmol of d7-cholesterol as an internal control. The samples were centrifuged at 10,000× *g* at 4 °C for 5 min. The supernatant fractions were collected and used for LC-MS and LC-MS/MS analysis, which were performed as described previously [18].

### 4.11. TEM Analysis

Subconfluent HeLa cell monolayers were generated by maintaining the cells in 2 mL of DMEM/10% FCS in 24-well plates. Next, the cells were infected with *C. trachomatis* for 2 h at an MOI of 2.0. The medium was replaced with DMEM/10% FCS containing 5 μM of (1*R*,3*R*)-HPA-12, 5 μM of E16A, or the vehicle solvent (DMSO). The chlamydia-infected cells were then maintained for 30 h. Next, the cells were washed three times with PBS and fixed at room temperature for 30 min with 1 mL of fixative solution (PBS containing 2.5% glutaraldehyde and 2% formaldehyde). The cells were then scraped with a rubber scraper and suspended in the fixative solution. Subsequently, the cell suspensions were incubated for 2 h at 4 °C and post-fixed in 1% osmium tetroxide. Next, the samples were dehydrated using a graded series of alcohol and propylene oxide. The samples were then embedded in an epoxy resin. Ultrathin sections were stained with uranyl acetate and lead citrate followed by observation under a transmission electron microscope (HT7700, Hitachi High Technologies, Tokyo, Japan) at 80 kV. The shape of *C. trachomatis* was classified into the following three groups: (1) EB, a small and black round-shaped spot; (2) RB, a gray spot; and (3) intermediate body (IB), a gray spot with a black center resembling a target.

## Data Availability

The original data used to prepare the figures in this paper are available at FigShare (doi:10.6084/m9.figshare.16534956).

## Supplementary Materials

The following supporting information can be downloaded at: https://www.mdpi.com/article/10.3390/ijms232314697/s1.
