# Supplementary material for: Chlamydial Infection-Dependent Synthesis of Sphingomyelin as a Novel Anti-Chlamydial Target of Ceramide Mimetic Compounds
Source: Int J Mol Sci. 2022 Nov 24;23(23):14697. doi: 10.3390/ijms232314697 (PMC9735676; doi:10.3390/ijms232314697)
Supplement: Supplementary file 1 [file ijms-23-14697-s001.zip › ijms-2005177-supplementary.pdf]

Supplementary Materials

Chlamydial Infection-Dependent Synthesis of Sphingomyelin as a Novel Anti-Chlamydial Target of Ceramide Mimetic Compounds

Keigo Kumagai [1,*], Shota Sakai [1], Masaharu Ueno [2], Michiyo Kataoka [3], Shu Kobayashi [4] and Kentaro Hanada [1,*,†]

[1] Department of Biochemistry and Cell Biology, National Institute of Infectious Diseases, 1-23-1 Toyama, Shinjuku-ku, Tokyo 162-8640, Japan

[2] Department of Natural Science, Graduate School of Advanced Technology and Science, Tokushima University, 2-1 Minami-jousanjima, Tokushima 770-8506, Japan

[3] Department of Pathology, National Institute of Infectious Diseases, 1-23-1 Toyama, Shinjuku-ku, Tokyo 162-8640, Japan

[4] Department of Chemistry, School of Science, the University of Tokyo, 7-3-1 Hongo, Bunkyo-ku, Tokyo 110-0033, Japan

* Correspondence: kuma@niid.go.jp (K.K.); hanak@niid.go.jp (K.H.)

† Present address: Department of Quality Assurance, Radiation Safety, and Information System, National Institute of Infectious Diseases, 1-23-1 Toyama, Shinjuku-ku, Tokyo 162-8640, Japan.

Figure S1. De novo SM synthesis in the presence of each compound.

Figure S2. Fluorescence images of HPA-12 stereoisomers separated by TLC.

Figure S3. Primary inclusion formation in *C. trachomatis* L2/434/Bu-infected cells grown in the presence of each compound.

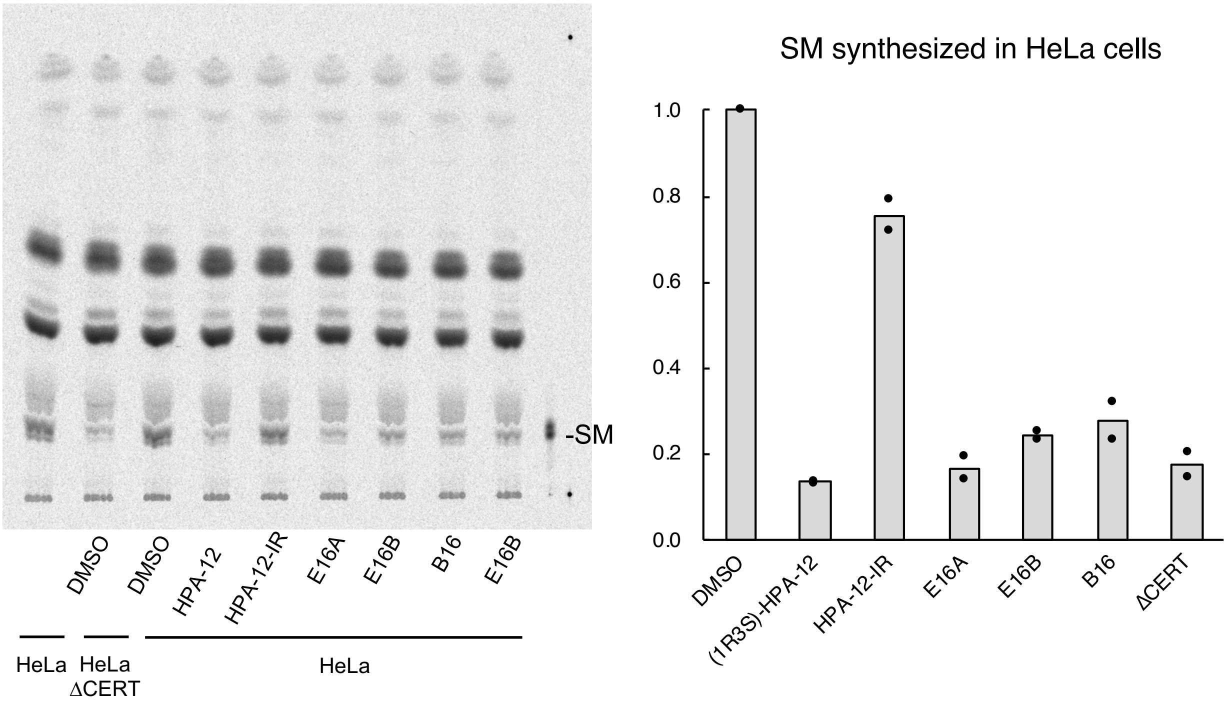

Figure S1. De novo SM synthesis in the presence of each compound. A, Confluent wild-type HeLa or HeLaΔCERT monolayers were cultured in SF-DMEM in 6-well plates. Next, the cells were labeled with 250 nCi of [14C]serine for 22 h in the presence of 3 μM of the indicated compound or DMSO. The lipids were extracted from the cells and separated by TLC. B, The ratios of the synthesized SM to the vehicle (DMSO) control are shown. The data are representative of two independent experiments. The values from each experiment are shown as dots and the averages are represented as bar graphs.

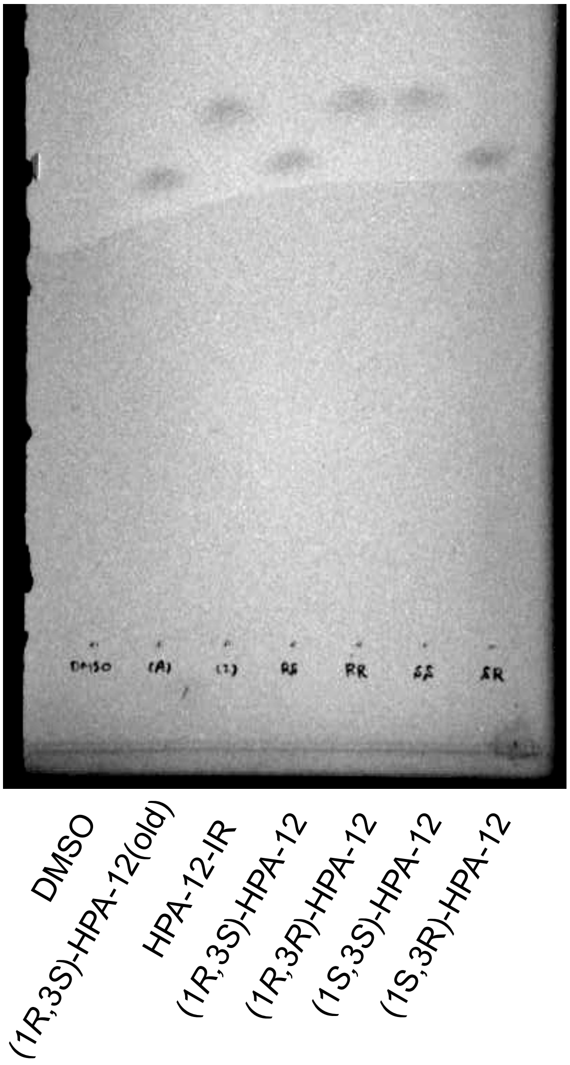

Figure S2. Fluorescence images of HPA-12 stereoisomers separated by TLC. The Silica gel 60 F254 TLC plate (Merck Millipore, Burlington, MA, USA) was prerinsed by blank chromatography with methanol and heated at 120˚C for 30 min to dehydrate the TLC plate. Twenty micrograms of each compound was spotted on the prerinsed TLC plate and separated using a solvent system (hexane/ethyl acetate = 30/60) from the bottom to the top with three iterations. "(1R,3S)-HPA-12 (old)" represents an old lot of (1R,3S)-HPA-12, which was chemically synthesized about 20 years ago along with the HPA-12-IR used in this study (16). The compounds were visualized with an AE-6905H Image Saver HR (Atto, Tokyo, Japan) with UV irradiation (254 nm). The (1R,3R)/(1S,3S) enantiomers with the "syn" orientation exhibited a larger retention factor (Rf) than the (1R,3S)/(1S,3R) enantiomers with the "anti" orientation.

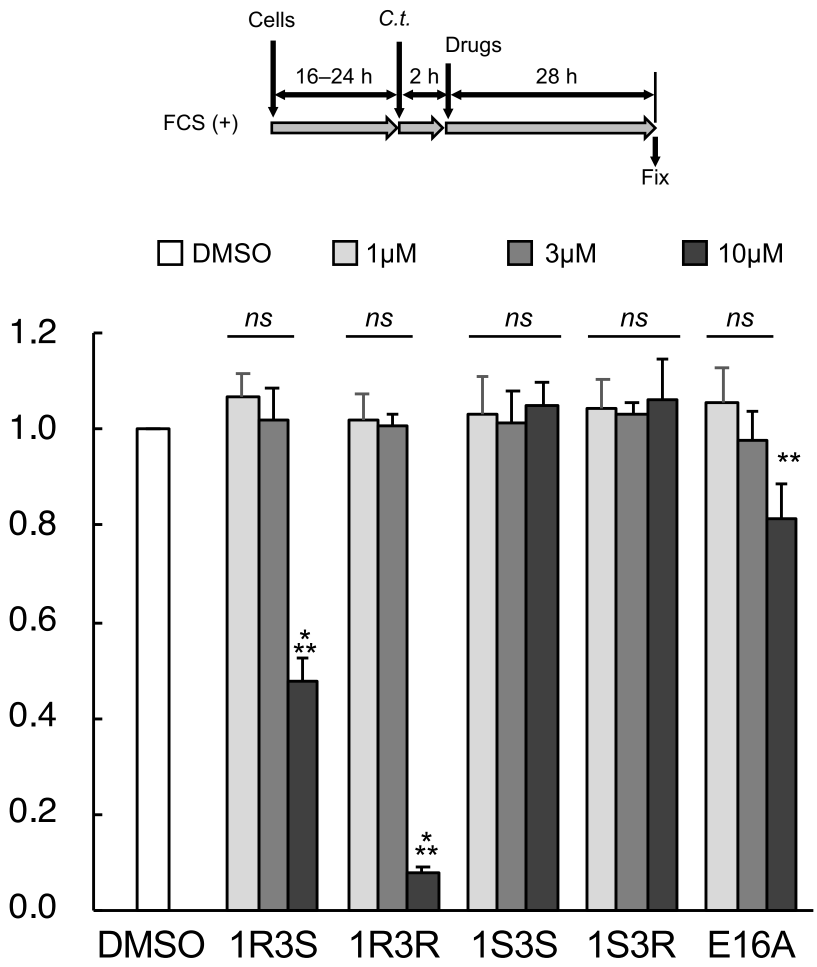

Figure S3. Primary inclusion formation in *C. trachomatis* L2/434/Bu-infected cells grown in the presence of each compound. The experimental procedures were identical to those described in the legend to Figure 6B, except that *C. trachomatis* L2/434/Bu was used as a pathogen. The statistically significant values assessed by Dunnett's test are indicated (**p<0.01; ***p<0.001; ns, not significant). The mean values ± SD from three experiments are shown (lower panel).
